# A composite measure of healthy lifestyle and the association with suicidal behaviors in Chinese young people aged 10–24 years: Results from a school-based survey in Zhejiang Province

**DOI:** 10.1371/journal.pone.0349662

**Published:** 2026-10-09

**Authors:** Meng Wang, Chun-Xiao Xu, Yun-Qi Guan, Yi-Fan Xu, Xiang-Yu Chen, Min Yu, Zhi-Min Ma, Jie-Ming Zhong

**Affiliations:** 1 Zhejiang Provincial Center for Disease Control and Prevention, Hangzhou, China; 2 University of Manchester, Manchester, England; Robert Gordon University, UNITED KINGDOM OF GREAT BRITAIN AND NORTHERN IRELAND

## Abstract

**Objectives:**

Smoking, alcohol drinking, dietary habits, physical activity, screen time, and sleep duration have been examined separately in relation to suicidality. Based on these behaviors, this study aimed to develop a composite measure of a healthy lifestyle, and to explore its associations with suicidal behaviors among Chinese young people aged 10–24 years.

**Methods:**

Data were obtained from 2022 Zhejiang Youth Risk Behavior Survey, in which 27,059 students aged 10–24 years completed an anonymous questionnaire designed to assess the health behaviors. A healthy lifestyle score was calculated and categorized into favorable, intermediate, and unfavorable lifestyle categories. Multivariate logistic regression models were employed to estimate the odds ratios (ORs) and 95% confidence intervals (CIs) for suicidal behaviors.

**Results:**

Overall, 21.12% of students followed a favorable lifestyle, 56.67% followed an intermediate lifestyle, and 22.21% followed an unfavorable lifestyle. Compared to unfavorable lifestyle, students with intermediate and favorable lifestyle had lower odds of suicidal ideation, with the OR was 0.67 (95% CI: 0.62–0.72) and 0.40 (0.36–0.45); for suicidal plans, the corresponding OR was 0.61 (0.55–0.67) and 0.37 (0.31–0.44); for suicidal attempts, the corresponding OR was 0.53 (0.45–0.62) and 0.27 (0.20–0.37).

**Conclusions:**

This study suggested that adherence to healthy lifestyles combining seven modifiable lifestyle factors were significantly associated with the lower odds of suicidal behaviors engagement in Chinese young people.

## Introduction

Suicide constitutes a global health crisis. Recent estimates from the World Health Organization (WHO) indicate that over 727,000 individuals died by suicide in 2021, yielding a global suicide rate of 9.2 per 100,000 population. Additionally, youth suicide remains a critical public health concern, ranking as the third leading cause of death among individuals aged 15–29 years [1]. Furthermore, existing evidence underscores that nonfatal suicidal behaviors, including ideation, plans, and attempts, are strong predictors of suicide mortality [2] and have emerged as a significant health issue among adolescents. Specifically, data from the Global School-based Health Survey (GSHS) revealed that the overall prevalence of suicidal ideation, plans, and attempts among adolescents aged 12–15 years across 83 countries was 16.5%, 16.5%, and 16.4%, respectively [3].

Various factors influence youth suicidality, including individual, family, and school-related elements such as gender, race/ethnicity, anxiety, feelings of loneliness, family conflict, lack of parental support, bullying, and school absenteeism [4–6]. Additionally, as individual behaviors typically develop during adolescence and persist into adulthood [7], their potential links to suicidality have attracted increasing attention. Substantial research has indicated that certain unhealthy individual behaviors, including cigarette smoking, alcohol drinking, insufficient physical activity, high sedentary behavior, short sleep duration, skipping breakfast and less fruit intake are positively associated with suicidal behaviors [8–12]. However, it is crucial to recognize that most of the aforementioned studies have primarily concentrated on single behaviors. Moreover, existing knowledge indicates that individual behaviors during adolescence are not isolated; they often co-occur in clustered patterns [13–15]. Consequently, it is insufficient to explore the effects of individual behavior on suicidality in isolation. Given these considerations, there is a pressing need to investigate how to integrate the primary behaviors into distinct behavioral patterns and to further understand how engagement in these patterns influences suicidal behaviors among young people.

Therefore, with the most recent data derived from a school-based survey of Chinese young people aged 10–24 years, this study attempted to develop a composite measure to express a healthy lifestyle and further to explore the possible effects on suicidal behaviors engagement.

## Materials and methods

### Participants

This study utilized a subset of the comprehensive dataset from the Zhejiang Youth Risk Behavior Survey (YRBS), a large-scale school-based survey conducted by the Zhejiang Provincial Center for Disease Control and Prevention (CDC). The YRBS study was initially designed to examine the health behaviors of Chinese students in 2007 and has been conducted every five years since. The study design and sampling strategy have been detailed in prior publication [16]. Briefly, this analysis employed data from the most recent survey round (the fourth), conducted between April 1, 2022, and June 29, 2022, utilizing multistage, stratified cluster sampling methods. Specifically, in the first stage, 30 counties (12 urban and 18 rural areas) were sampled from all 90 counties of Zhejiang province on the basis of socioeconomic status. The 12 urban areas included Shangcheng, Xiacheng, Haishu, Lucheng, Putuo, Kecheng, Wuxing, Nanhu, Wucheng, Yuecheng, Jiaojiang, and Liandu. The 18 rural areas included Fuyang, Jiande, Yuyao, Fenghua, Jiashan, Tongxiang, Yueqing, Pingyang, Xianju, Wenling, Shangyu, Xinchang, Wuyi, Dongyang, Anji, Kaihua, Suichang, and Yunhe. Then, all schools in selected counties were stratified according to their levels (middle school, academic and vocational high school) and geographical positions (from west to east, from north to south). In the second stage, classes were selected from each educational level based on the number of students enrolled. Finally, students from all chosen classes were invited to participate in the survey. A total of 27,070 middle and high school students from 706 classes across 376 schools in 30 counties were recruited, yielding a response rate of 96.53%. After excluding a small number of students with missing key information regarding age (n = 9) and gender (n = 2), 25,059 students remained for this analysis. Data on demographic characteristics and health behaviors-including physical activity, dietary habits, cigarette smoking, alcohol drinking, sleep duration, screen time and other behaviors were collected using a self-administered questionnaire adapted from the US 1991−2015 Youth Risk Behavior Surveillance System (YRBSS) and the GSHS. Students independently completed the anonymous questionnaire in the classroom, after which the researchers collected the questionnaires on site. To ensure that participation was entirely voluntary, written letters were sent to the parents or guardians of the students, as well as to school officials, providing them with the option to refuse participation. The letters also included a request for consent to publish the collected data without any identifying information. Furthermore, researchers underwent rigorous training to safeguard the students’ privacy and maintain the confidentiality of personal data. This study abided by the “Declaration of Helsinki” and was approved by the ethics committee of Zhejiang CDC (2022-007-01).

### Outcome assessment

Suicidal behaviors, encompassing ideation, plans and attempts, constituded the primary outcome of this study. These behaviors were assessed through the following question: “Have you seriously considered suicide in the last 12 months?” “Have you made a specific plan to commit suicide in the last 12 months?” and “Have you attempted suicide in the last 12 months?” Respondents answered each question with either “yes” or “no”.

### Healthy lifestyle composite score and categories

In this study, a healthy lifestyle score was generated based on the previous reported suicidal behaviors related factors: smoking, alcohol drinking, physical activity, screen time, fruits and vegetables intake, breakfast consumption, and sleep duration. Students scored one point for each of the seven health behaviors according to the recommended guidance with the following questionnaire items and response options. It should be noted that this approach is widely adopted in constructing composite lifestyle indices as it facilitates interpretability, clinical utility, and risk communication [17–19]. Students were asked to report the number of days they have smoked cigarettes and drank alcohol in the past 30 days. The options were “0 day”, “1-2 days”, “3-5 days”, “6-9 days”, “10-19 days”, “20-29 days”, “30 days”. Participants who have chosen “0 day” for smoking and alcohol drinking were considered to have the never smoking (one point) [20] and never drinking (one point) [21] behavior, respectively. Students were asked to report the number of days they have participated in at least 60 minutes of any kind of physical activity that increased heart rate and made breathe hard during the past seven days. The options were “0 day”, “1 day”, “2 days”, “3 days”, “4 days”, “5 days”, “6 days”, “7 days”. Based on responses, engaging in physical activity was dichotomized into daily (one point) and non-daily [22]. Students were asked to indicate the customary time that they spend watching TV, playing video games and using the computer, with the options were “never”, “<1 hour per day”, “1-2 hours per day”, “2-3 hours per day”, “3-4 hours per day”, “4-5 hours per day”, “≥5 hours per day”. Screen time was calculated by the sum of these behaviors and was dichotomized into <2 hours (one point) and ≥2 hours per day [23]. Students asked to report the number of days they have had their breakfast in the past seven days, with answers were given on an 8-point scale (1 = none to 8 = 7days). Based on these answers, breakfast consumption behavior was dichotomized into daily (one point) and non-daily [24]. Students were asked to report the frequency that they consumed fruits and vegetables per day during the past month. The options were “never”, “<1 time per day”, “1 time per day”, “2 times per day”, “3 times per day”, “4 times per day”, “≥5 times per day”. Participants who have answered “1 time per day or more” were considered to have the behavior of fruits / vegetables intake daily (one point) [25]. Students were asked to indicate the customary sleep time per day during the past month and the sleep duration was dichotomized into <8 hours and ≥8 hours per day (one point) [26]. The healthy lifestyle scores ranged from zero to seven, with the higher score indicating healthier lifestyles, and were further categorized as unfavorable lifestyle (zero to three healthy lifestyle scores), intermediate lifestyle (four to five healthy lifestyle scores), and favorable lifestyle (six to seven healthy lifestyle scores). To assess the validity of the healthy lifestyle scores and categories, a weighted standardized healthy lifestyle score (ranging from 0 to 100) was also employed. This score was derived from β coefficients and the average proportion of each lifestyle factor identified in binary logistic regression models related to suicidal ideation, plans and attempts, incorporating all seven lifestyle factors while adjusting for relevant socio-demographic and mental health-related confounding variables. Specifically, we calculated the proportion of each of the seven lifestyle factors based on the β coefficients obtained from the models addressing suicidal ideation, plans and attempts, followed by the calculation of the average proportion for each factor. The original binary lifestyle variables were multiplied by their corresponding average proportions, summed, and then multiplied by 100. The resulting weighted standardized lifestyle score was categorized as favorable, intermediate, or unfavorable according to the distribution of the primary healthy lifestyle scores.

### Statistical analysis

Descriptive statistics were employed to characterize students based on suicidal behaviors, including ideation, plans, and attempts. The characteristics of students exhibiting suicidal behavior were compared to those without such behaviors using Kruskal-Wallis test for continuous variables and the linear-by-linear association χ^2^-test for categorical variables. Multivariate logistic regression models were utilized to estimate odds ratios (ORs) with 95% confidence intervals (CIs) for the associations between suicidal behaviors (ideation, plans and attempts) and the individual healthy lifestyle factors, as well as healthy lifestyle scores and categories. Adjustment for confounding factors was performed using three sequential models. Model 1, we adjusted for socio-demographic characteristics of age and gender. Model 2 was further adjusted for location of school, school type, school performance, paternal education, maternal education, parental marriage status, and body mass index. Model 3 accounted for all variables in model 2, along with mental health-related characteristics, including feelings of loneliness, sleep loss due to worry and experiences of sadness or despair. To assess the validity of the healthy lifestyle categories, we conducted the same analyses across the three models for the weighted healthy lifestyle caterogies. Furthermore, to examine how the OR varies with the inclusion of each factor in the combined score, we presented estimates of suicidal ideation, plans, and attempts for specific lifestyle patterns within each score category, both for the overall student population and for those exhibiting suicidal ideation, plans, and attempts. All analyses were performed using SAS statistical package (version 9.4, SAS Institute, Inc., Cary, NC, USA). All statistical tests were based on the two-sided 5% level of significance.

## Results

Table 1 presented the basic characteristics of the study participants. A total of 27,059 young people aged 10–24 years were enrolled into this analysis, including 5229 (19.32%) students with suicidal ideation, 2284 (8.44%) with suicidal plans, and 799 (2.95%) with suicidal attempts. Nearly half of the students were boys (51.59%) and from middle school (47.30%). The majority of students were from rural school (59.13%) and reported that parents had the middle school or below education level (paternal 55.32% and maternal 59.16%). About a third of the students reported their school performance as below average (30.52%). Few students were obese (3.80%), or current smokers (4.14%). Compared to students without any suicidal behavior, those with suicidal behaviors tended to be younger, girls, from middle school, with below average performance but higher paternal / maternal education, and have higher proportion of divorced / separated / widowed of parental marriage status, mental health-related characteristics, smoking, alcohol drinking, non-daily consumption of fruits / vegetables and breakfast, longer screen time and shorter sleep duration in each day, and fewer number of healthy lifestyle factors. Most students reported either four or five (56.67%) of seven healthy lifestyle factors (intermediate healthy lifestyle), while 22.21% and 21.12% of students reported less than four (unfavorable healthy lifestyle) and more than five (favorable healthy lifestyle) healthy lifestyle factors, respectively. For the weighted lifestyle score, 25.77% were categorized as following an unfavorable lifestyle (scores ranging from 0 to 54), 55.76% followed an intermediate lifestyle (scores ranging 55–87), and 18.47% followed a favorable lifestyle (scores ranging 88–100).

**Table 1:** Characteristics of study participants in Zhejiang Province according to suicidal ideation, plans, and attempts.

Characteristics	Overall (27,059)	Suicidal ideation (5229)	Suicidal Plans (2284)	Suicidal Attempts (799)	No suicidal behaviors (21,487)	P value‡
Social-demographic characteristics						
Mean age at survey, years	15.75	15.45	15.37	15.11	15.83	<0.001
Boys, %	51.59	38.15	36.12	25.66	54.94	<0.001
Urban school, %	40.87	40.62	40.37	39.05	40.87	0.847
Middle school level, %	47.30	55.27	57.79	64.58	45.19	<0.001
School performance (below average), %	30.52	38.92	41.22	44.92	28.41	<0.001
Middle school or below (paternal), %	55.32	54.54	54.84	54.32	55.55	<0.001
Middle school or below (maternal), %	59.16	58.48	59.26	59.07	59.27	<0.001
Parental marriage status (divorced / separated / widowed), %	12.93	16.90	18.18	22.03	11.90	<0.001
Obesity, %	3.80	4.05	7.84	4.88	3.37	<0.001
Mental health-related characteristics, %						
Feelings of loneliness	78.06	95.33	94.09	97.63	73.79	<0.001
Sleep loss due to worry	66.69	85.70	87.87	92.74	61.90	<0.001
Experience of sadness / despair	17.69	43.55	51.34	66.17	11.31	<0.001
Healthy lifestyle factors, %						
Never smoking	95.86	93.84	92.19	87.84	96.40	<0.001
Never drinking	83.54	76.57	73.33	63.35	85.22	<0.001
Physical activity daily	12.86	13.04	14.49	15.52	12.80	0.504
Screen time < 2 h per day	58.11	55.56	52.69	46.43	58.80	<0.001
Fruits / vegetables intake daily	68.26	61.55	60.42	64.08	69.90	<0.001
Breakfast consumption daily	69.32	55.90	51.40	44.18	72.94	<0.001
Sleep duration ≥ 8 h per day	56.31	43.32	41.99	39.80	59.58	<0.001
Mean No. of healthy lifestyle factors	4.45	4.00	3.87	3.62	4.56	<0.001

‡ For continuous and categorical variables, difference in characteristics of students with or without any suicidal behavior was compared using Kruskal-Wallis test and linear-by-linear association χ2-test, respectively.

Percentages are calculated based on the sample size of each column. Absolute number of participants can be obtained by multiplying percentages by the sample size of each column.

Table 2 showed the main effect of individual healthy lifestyle factor on suicidal ideation, plans, and attempts, respectively. Among the study participants, after adjusting for the potential confounding factors, never smoking, as well as never drinking, screen time < 2 h per day, fruits / vegetables intake daily, breakfast consumption daily and sleep duration ≥ 8 h per day were associated with statistically significant decreased odds of suicidal behaviors, whereas physical activity daily was related to significant increased odds.

**Table 2:** Adjusted odds ratios (95% CIs) of suicidal ideation, plans, and attempts by the individual healthy lifestyle factors among Zhejiang students.

Healthy lifestyle factors (yes vs. no)	Cases / total	Model 1	Model 2	Model 3
**Suicidal ideation**				
Never smoking	4903/25,900	0.43 (0.38-0.50)	0.43 (0.38-0.50)	0.64 (0.55-0.75)
Never drinking	3987/22,512	0.49 (0.45-0.52)	0.48 (0.44-0.52)	0.66 (0.61-0.72)
Physical activity daily	682/3479	1.13 (1.03-1.24)	1.10 (1.01-1.21)	1.12 (1.01-1.24)
Screen time < 2 h per day	2904/15,708	0.84 (0.79-0.89)	0.83 (0.77-0.88)	0.95 (0.88-0.96)
Fruits / vegetables intake daily	3217/18,452	0.64 (0.60-0.68)	0.62 (0.58-0.67)	0.68 (0.64-0.73)
Breakfast consumption daily	2921/18,736	0.49 (0.46-0.52)	0.50 (0.47-0.53)	0.63 (0.59-0.68)
Sleep duration ≥ 8 h per day	2265/15,237	0.51 (0.48-0.54)	0.50 (0.47-0.54)	0.63 (0.59-0.67)
**Suicidal plans**				
Never smoking	2102/25,898	0.35 (0.29-0.41)	0.35 (0.29-0.42)	0.54 (0.44-0.65)
Never drinking	1639/22,510	0.43 (0.39-0.48)	0.43 (0.38-0.47)	0.61 (0.54-0.68)
Physical activity daily	331/3478	1.30 (1.14-1.47)	1.26 (1.10-1.43)	1.24 (1.08-1.43)
Screen time < 2 h per day	1203/15,708	0.74 (0.68-0.81)	0.77 (0.70-0.84)	0.90 (0.81-0.98)
Fruits / vegetables intake daily	1380/18,451	0.64 (0.58-0.69)	0.60 (0.55-0.66)	0.67 (0.61-0.74)
Breakfast consumption daily	1173/18,736	0.45 (0.41-0.49)	0.51 (0.47-0.56)	0.68 (0.61-0.74)
Sleep duration ≥ 8 h per day	959/15,236	0.52 (0.48-0.57)	0.49 (0.45-0.54)	0.64 (0.58-0.70)
**Suicidal attempts**				
Never smoking	701/25,899	0.18 (0.14-0.22)	0.19 (0.15-0.25)	0.32 (0.24-0.41)
Never drinking	503/22,511	0.26 (0.22-0.30)	0.27 (0.23-0.31)	0.40 (0.34-0.48)
Physical activity daily	124/3479	1.50 (1.23-1.84)	1.40 (1.14-1.71)	1.34 (1.09-1.66)
Screen time < 2 h per day	371/15,707	0.56 (0.49-0.65)	0.60 (0.51-0.69)	0.71 (0.61-0.84)
Fruits / vegetables intake daily	512/18,452	0.74 (0.64-0.86)	0.72 (0.62-0.84)	0.84 (0.72-0.99)
Breakfast consumption daily	353/18,736	0.36 (0.31-0.41)	0.38 (0.33-0.45)	0.54 (0.47-0.63)
Sleep duration ≥ 8 h per day	318/15,236	0.50 (0.43-0.58)	0.47 (0.40-0.54)	0.67 (0.57-0.78)

Model 1 adjusted for age and gender; model 2 adjusted for model 1 plus location of school, school type, school performance, paternal education, maternal education, parental marriage status, and body mass index; model 3 adjusted for model 2 plus mental health-related characteristics of feelings of loneliness, sleep loss due to worry and experience of sadness / despair.

Table 3 showed the composite healthy lifestyle scores and categories in relation to suicidal ideation, plans, and attempts, respectively. After adjusting for the potential confounding factors, compared to the least healthy score (0 point), a 57−82% reduction (ORs ranged from 0.18 to 0.43 and the 95% CIs did not include the null) in odds of suicidal ideation was observed in the combination of three to seven healthy lifestyle factors (3–7 points); compared to unfavorable lifestyle, students with intermediate and favorable lifestyle had a reduced odds of suicidal ideation, with the OR was 0.67 (95% CI: 0.62–0.72) and 0.40 (0.36–0.45), respectively. For suicidal plans, although non-significant relationships with each composite healthy lifestyle score were observed, students with intermediate and favorable lifestyle had a reduced odds of suicidal plans, with the OR was 0.61 (0.55–0.67) and 0.37 (0.31–0.44), respectively. Regarding to suicidal attempts, compared to the least healthy score (0 point), a 68−87% reduction (ORs ranged from 0.13 to 0.32 and the 95% CIs did not include the null) in odds was observed in the combination of four to seven healthy lifestyle factors (4–7 points); compared to unfavorable lifestyle, students with intermediate and favorable lifestyle had a reduced odds of suicidal attempts, with the OR was 0.53 (0.45–0.62) and 0.27 (0.20–0.37), respectively. When the same analyses were conducted for the weighted lifestyle categories, the results were largely unchanged (S1 Table).

**Table 3:** Adjusted odds ratios (95% CIs) of suicidal ideation, plans, and attempts by the healthy lifestyle scores and categories among Zhejiang students.

Healthy lifestyle scores and categories	Cases / total	Model 1	Model 2	Model 3
**Suicidal ideation**				
Healthy lifestyle scores				
0	23/49	1.00 (Ref.)	1.00 (Ref.)	1.00 (Ref.)
1	152/377	0.72 (0.39-1.34)	0.67 (0.35-1.25)	0.70 (0.36-1.38)
2	524/1572	0.49 (0.27-0.88)	0.44 (0.24-0.80)	0.58 (0.30-1.10)
3	1010/3980	0.32 (0.18-0.57)	0.28 (0.15-0.51)	0.43 (0.26-0.81)
4	1574/7062	0.26 (0.14-0.46)	0.23 (0.12-0.41)	0.38 (0.20-0.72)
5	1302/8189	0.16 (0.09-0.29)	0.14 (0.08-0.25)	0.27 (0.14-0.51)
6	533/4815	0.10 (0.06-0.18)	0.08 (0.05-0.15)	0.19 (0.10-0.36)
7	80/870	0.09 (0.05-0.16)	0.07 (0.04-0.13)	0.18 (0.09-0.36)
P for trend		<0.001	<0.001	<0.001
Healthy lifestyle categories				
Unfavorable	1709/5978	1.00 (Ref.)	1.00 (Ref.)	1.00 (Ref.)
Intermediate	2876/15,251	0.54 (0.50-0.57)	0.53 (0.49-0.57)	0.67 (0.62-0.72)
Favorable	613/5685	0.26 (0.24-0.29)	0.25 (0.22-0.28)	0.40 (0.36-0.45)
**Suicidal plans**				
Healthy lifestyle scores				
0	7/49	1.00 (Ref.)	1.00 (Ref.)	1.00 (Ref.)
1	86/377	1.76 (0.75-4.12)	1.71 (0.72-4.07)	1.87 (0.77-4.56)
2	261/1571	1.08 (0.47-2.47)	1.01 (0.43-2.34)	1.41 (0.59-3.33)
3	483/3979	0.72 (0.32-1.64)	0.69 (0.30-1.60)	1.13 (0.48-2.67)
4	653/7062	0.51 (0.22-1.15)	0.48 (0.21-1.11)	0.87 (0.37-2.04)
5	512/8189	0.32 (0.14-0.73)	0.30 (0.13-0.69)	0.65 (0.27-1.53)
6	194/4815	0.19 (0.08-0.44)	0.17 (0.07-0.40)	0.44 (0.19-1.06)
7	33/870	0.19 (0.08-0.46)	0.17 (0.07-0.42)	0.50 (0.20-1.26)
P for trend		<0.001	<0.001	<0.001
Healthy lifestyle categories				
Unfavorable	837/5976	1.00 (Ref.)	1.00 (Ref.)	1.00 (Ref.)
Intermediate	1165/15,251	0.47 (0.43-0.52)	0.47 (0.42-0.52)	0.61 (0.55-0.67)
Favorable	227/5685	0.22 (0.19-0.26)	0.21 (0.18-0.25)	0.37 (0.31-0.44)
**Suicidal attempts**				
Healthy lifestyle scores				
0	6/49	1.00 (Ref.)	1.00 (Ref.)	1.00 (Ref.)
1	49/377	1.05 (0.41-2.69)	1.02 (0.39-2.69)	1.03 (0.38-2.76)
2	126/1572	0.52 (0.21-1.29)	0.51 (0.20-1.30)	0.68 (0.26-1.76)
3	171/3980	0.25 (0.10-0.63)	0.26 (0.10-0.66)	0.42 (0.16-1.07)
4	220/7061	0.17 (0.07-0.42)	0.18 (0.07-0.45)	0.32 (0.13-0.82)
5	162/8189	0.10 (0.04-0.25)	0.10 (0.04-0.26)	0.24 (0.09-0.61)
6	49/4815	0.05 (0.02-0.13)	0.05 (0.02-0.12)	0.13 (0.05-0.36)
7	10/870	0.06 (0.02-0.18)	0.06 (0.02-0.17)	0.19 (0.06-0.60)
P for trend		<0.001	<0.001	<0.001
Healthy lifestyle categories				
Unfavorable	352/5978	1.00 (Ref.)	1.00 (Ref.)	1.00 (Ref.)
Intermediate	382/15,250	0.37 (0.32-0.43)	0.38 (0.32-0.44)	0.53 (0.45-0.62)
Favorable	59/5685	0.14 (0.11-0.19)	0.14 (0.10-0.18)	0.27 (0.20-0.37)

Model 1 adjusted for age and gender; model 2 adjusted for model 1 plus location of school, school type, school performance, paternal education, maternal education, parental marriage status, and body mass index; model 3 adjusted for model 2 plus mental health-related characteristics of feelings of loneliness, sleep loss due to worry and experience of sadness / despair.

In this study, there are 128 healthy lifestyle patterns derived from different combinations of the seven lifestyle factors. For simplicity, based on the overall lifestyle factor proportion among overall participants (Table 1), we presented the certain pattern in each score category, including the factor and order (Table 4). The only combination of never smoking and never drinking behaviors was not significantly associated with the odds of suicidal ideation, plans and attempts. The odds of suicidal ideation and attempts decreased with the addition of each healthy lifestyle factor in the following order: breakfast consumption daily, fruits and vegetables intake daily, screen time < 2h per day, sleep duration ≥ 8h per day, and physical activity daily.

**Table 4:** Adjusted odds ratios (95% CIs) of suicidal ideation, plans, and attempts for the most common healthy lifestyle pattern distributed among overall Zhejiang students.

Healthy lifestyle scores	No. of cases	Score for healthy lifestyle factor							Suicidal ideationǂ	Suicidal plansǂ	Suicidal attemptsǂ
		Never smoking	Never drinking	Physical activity daily	Screen time < 2 h per day	Fruits /vegetables intake daily	Breakfast consumption daily	Sleep duration ≥ 8 h per day			
0	49	0	0	0	0	0	0	0	1.00 (Ref.)	1.00 (Ref.)	1.00 (Ref.)
1	185	1	0	0	0	0	0	0	1.02 (0.50-2.09)	2.47 (0.97-6.26)	1.12 (0.38-3.31)
2	532	1	1	0	0	0	0	0	0.66 (0.33-1.29)	1.64 (0.67-4.00)	0.40 (0.14-1.17)
3	655	1	1	0	0	0	1	0	0.41 (0.21-0.81)	1.05 (0.42-2.61)	0.09 (0.02-0.33)
4	1162	1	1	0	0	1	1	0	0.34 (0.17-0.65)	0.81 (0.33-1.97)	0.30 (0.11-0.86)
5	2738	1	1	0	1	1	1	0	0.32 (0.17-0.63)	0.82 (0.34-1.98)	0.20 (0.07-0.57)
6	3675	1	1	0	1	1	1	1	0.19 (0.10-0.36)	0.45 (0.18-1.10)	0.11 (0.04-0.31)
7	870	1	1	1	1	1	1	1	0.20 (0.10-0.40)	0.64 (0.25-1.64)	0.20 (0.06-0.65)

ǂ The effect values were based on model 3, which adjusted for all the socio-demographic characteristics and mental health-related characteristics.

## Discussion

With the data from a large-scale school-based survey among Chinese young people aged 10–24 years old, our findings confirmed that engagement in specific health-promoting behaviors, such as never smoking, never drinking, less screen time, daily consumption of fruits and vegetables and breakfast, and longer sleep duration was significantly associated with a reduced likelihood of suicidal behaviors. Besides, the results indicated that participants exhibiting a greater number of healthy lifestyle factors had lower odds of suicidal behaviors, demonstrating a significant trend. When categorized into distinct lifestyle patterns, the results revealed that participants adhering to intermediate and favorable lifestyles had reduced odds of suicidal behaviors compared to those following unfavorable lifestyles. To our knowledge, this is one of the few studies attempting to generate a composite healthy lifestyle and to examine the associations with suicidal behaviors among Chinese young people.

This study indicated that young individuals who adhered to intermediate and favorable lifestyles exhibited a lower likelihood of engaging in suicidal behaviors compared to those who followed unfavorable lifestyle. These findings largely align with results from prior research. For example, with data derived from a cross-national study of the GSHS between 2009 and 2019, the authors [27] used latent class analysis (LCA) method and constructed four lifestyle clusters among 229,041 adolescents, namely healthy lifestyle (H-L), insufficient intake of vegetables and fruit (V-F), frequent consumption of soft drinks and fast food (S-F), and tobacco smoking and alcohol drinking (S-A). After analysis, the results showed that compared with the H-L cluster, V-F and S-A clusters were associated with increased odds of suicidal ideation, plans and attempts. Similarly, using the same method of LCA, a Chinese study of 5291 middle school students in Guangxi Province [28] identified three classes lifestyles (high risk, high lack of exercise and low risk) and found that students among the high risk lifestyle group had higher odds of suicide-related behaviors. Besides, Xiao et al. [29] also established three healthy lifestyles with 13 behaviors among a US nationally representative sample of high school students, and further analysis results equally suggested that compared to the consistent engagement in health-promoting behaviors, individuals with the lowest engagement were associated with higher odds of suicidal plans. However, notably, this study reported that students with moderate engagement in health-promoting behaviors had much lower odds of suicidal attempts, relative to the consistent engagement group, which called for more research to focus on the specific levels of engagement in health-promoting behaviors that could induce beneficial effects.

In addition, the analysis of a specific healthy lifestyle pattern among the study participants revealed that while never smoking and never drinking exhibited relatively stronger effects as individual lifestyle factors, the combination of these two behaviors did not render reduced odds of suicidal behaviors. Interestingly, we noted that the inclusion of daily consumption of breakfast along with other healthy lifestyle factors, began to yield reductions in odds. Notably, most epidemiological studies have demonstrated that engagement in physical activity may serve as an effective strategy for the prevention of suicidal behaviors [30–32]. Conversely, findings from the limited prior studies, along with our own research, indicate that individual engagement in physical activity is positively associated with suicidal behaviors [33–35]. Nevertheless, we found that incorporating daily physical activity into the predominant lifestyle patterns among young people significantly reduced the odds of suicidal ideation and attempts. These observations may reflect the interactions among various healthy lifestyle factors and underscore the importance of researching specific healthy lifestyle patterns.

The strengths of this study included data derived from a recent and relatively large survey encompassing a wide range of healthy behaviors, extensive adjustments for potential confounders in the analysis, and an examination of the associations between healthy lifestyle patterns and suicidal behaviors among Chinese young people. However, the current study also had some limitations. Firstly, due to its cross-sectional design, it is not possible to infer causality regarding the reported associations between healthy lifestyles and suicidal behaviors without longitudinal data. Secondly, the participant information was collected through self-administered questionnaires, which may introduce reporting bias for the lifestyle factors, suicidal behaviors and other covariates analyzed. Besides, the time frames used to assess individual lifestyle factors (ranging from one week to one month) were shorter than the duration for suicidal behaviors (12 months) as reported in the questionnaire, potentially leading to temporal bias in the analysis and an overestimation of these associations. Thirdly, since the study sample was selected at the provincial level, these findings may not be generalizable to all young people in China.

## Conclusions

In conclusion, this study suggested that the combined healthy lifestyle patterns, with the components of never smoking, never drinking, less screen time, daily consumption of fruits and vegetables and breakfast, and longer sleep duration were associated with lower odds of suicidal behaviors among Chinese young people. These findings had several real-world implications in suicidality prevention. Firstly, school health professionals should adopt an integrated approach that targets these behaviors as a cluster, rather than addressing the individual behaviors. Secondly, schools with limited prevention resources can prioritize interventions focusing on this set of behaviors, particularly ensuing the daily consumption of breakfast, which may serve as a foundational factor. Thirdly, the present and more identification of behavioral combination can provide practical framework for developing screening tools, enable school counselors and nurses to identify at-risk individuals earlier through routine health assessments. Although further searching for suicidal-related factors may contribute to innovative preventive strategies, school health professionals and researchers should realize and consider to incorporate the possible effects of combined healthy lifestyles aforementioned into developing of educational programs on suicidality prevention among school students.

## Supporting information

S1 Table Adjusted odds ratios (95% CIs) of suicidal ideation, plans, and attempts by the weighted healthy lifestyle scores and categories among Zhejiang students. (DOCX)
