# Supplementary material for: A composite measure of healthy lifestyle and the association with suicidal behaviors in Chinese young people aged 10–24 years: Results from a school-based survey in Zhejiang Province
Source: PLoS One. 2026 Oct 9;21(10):e0349662. doi: 10.1371/journal.pone.0349662 (PMC13652226; doi:10.1371/journal.pone.0349662)
Supplement: Supplementary file 1 [file pone.0349662.s001.docx]

| Table S1. Adjusted odds ratios (95% CIs) of suicidal ideation, plans, and attempts by the weighted healthy lifestyle scores and categories among Zhejiang students. | | | | |
| --- | --- | --- | --- | --- |
| Weighted healthy lifestyle categories | Cases / total | Model 1 | Model 2 | Model 3 |
| **Suicidal ideation** |  |  |  |  |
| Weighted healthy lifestyle categories |  |  |  |  |
| Unfavorable (ranging from 0 to 54) | 2165/7451 | 1.00 (Ref.) | 1.00 (Ref.) | 1.00 (Ref.) |
| Intermediate (ranging 55 to 87) | 2531/14,342 | 0.49 (0.46-0.53) | 0.49 (0.46-0.53) | 0.62 (0.58-0.67) |
| Favorable (ranging 88 to 100) | 502/5121 | 0.23 (0.21-0.26) | 0.22 (0.20-0.25) | 0.36 (0.32-0.41) |
| **Suicidal plans** |  |  |  |  |
| Weighted healthy lifestyle categories |  |  |  |  |
| Unfavorable (ranging from 0 to 54) | 1023/7449 | 1.00 (Ref.) | 1.00 (Ref.) | 1.00 (Ref.) |
| Intermediate (ranging 55 to 87) | 1030/14,342 | 0.46 (0.42-0.50) | 0.47 (0.42-0.51) | 0.61 (0.55-0.68) |
| Favorable (ranging 88 to 100) | 176/5121 | 0.20 (0.17-0.23) | 0.20 (0.17-0.24) | 0.35 (0.29-0.41) |
| **Suicidal attempts** |  |  |  |  |
| Weighted healthy lifestyle categories |  |  |  |  |
| Unfavorable (ranging from 0 to 54) | 426/7451 | 1.00 (Ref.) | 1.00 (Ref.) | 1.00 (Ref.) |
| Intermediate (ranging 55 to 87) | 318/14,341 | 0.35 (0.30-0.40) | 0.36 (0.31-0.42) | 0.51 (0.44-0.60) |
| Favorable (ranging 88 to 100) | 49/5121 | 0.14 (0.10-0.18) | 0.14 (0.10-0.18) | 0.28 (0.20-0.38) |
| Model 1 adjusted for age and gender; model 2 adjusted for model 1 plus location of school, school type, school performance, paternal education, maternal education, parental marriage status, and body mass index; model 3 adjusted for model 2 plus mental health-related characteristics of feelings of loneliness, sleep loss due to worry and experience of sadness / despair. | | | | |
